# Combining Dispersion Modeling and Monitoring Data for Community-Scale Air Quality Characterization

**DOI:** 10.3390/atmos10100610

**Published:** 2019

**Authors:** Vlad Isakov, Saravanan Arunachalam, Richard Baldauf, Michael Breen, Parikshit Deshmukh, Andy Hawkins, Sue Kimbrough, Stephen Krabbe, Brian Naess, Marc Serre, Alejandro Valencia

**Affiliations:** 1 Office of Research and Development, U.S. EPA, Research Triangle Park, NC 27711, USA; 2 Institute for the Environment, University of North Carolina at Chapel Hill, Chapel Hill, NC 27517, USA; 3 Office of Transportation and Air Quality, U.S. EPA, Ann Arbor, MI 48105, USA; 4 Jacobs Technology, Research Triangle Park, NC 27711, USA; 5 U.S. EPA, Region 7, Kansas City, KS 66219, USA; 6 Department of Environmental Sciences and Engineering, University of North Carolina at Chapel Hill, Chapel Hill, NC 27599, USA

**Keywords:** near-source, dispersion modeling, rail yard, air pollution

## Abstract

Spatially and temporally resolved air quality characterization is critical for community-scale exposure studies and for developing future air quality mitigation strategies. Monitoring-based assessments can characterize local air quality when enough monitors are deployed. However, modeling plays a vital role in furthering the understanding of the relative contributions of emissions sources impacting the community. In this study, we combine dispersion modeling and measurements from the Kansas City TRansportation local-scale Air Quality Study (KC-TRAQS) and use data fusion methods to characterize air quality. The KC-TRAQS study produced a rich dataset using both traditional and emerging measurement technologies. We used dispersion modeling to support field study design and analysis. In the study design phase, the presumptive placement of fixed monitoring sites and mobile monitoring routes have been corroborated using a research screening tool C-PORT to assess the spatial and temporal coverage relative to the entire study area extent. In the analysis phase, dispersion modeling was used in combination with observations to help interpret the KC-TRAQS data. We extended this work to use data fusion methods to combine observations from stationary, mobile measurements, and dispersion model estimates.

## 1. Introduction

An increasing number of health studies have revealed associations between proximity to major transportation hubs and adverse public health effects [1]. People who live, work, or attend school near major roads, ports or rail yards have an increased incidence and severity of health problems associated with air pollution exposures related to mobile sources. Because many of the major transportation hubs are located near disadvantaged and lower-income communities, it also raises environmental justice issues. Many communities located near major transportation sources such as freeways, rail yards, distribution centers, ports, and airports, typically have higher levels of air pollution than other areas [2–4]. Industrial facilities can also contribute to air pollution exposure. Thus, communities could experience cumulative impacts from exposure to multiple air pollutants due to emissions from mobile sources such as cars, trucks and locomotives, and other stationary sources located within or in the proximity of neighborhoods. Community groups are becoming increasingly active in local initiatives that seek to mitigate potentially harmful environmental conditions. Therefore, spatially and temporally resolved air quality characterization is critical for community-scale exposure studies and for developing future air quality mitigations. First, spatially resolved air quality characterization is needed to identify areas with elevated levels of pollutant concentrations. Then, understanding relative contributions of emissions sources impacting the community is necessary for developing mitigation strategies. Various air quality modelling approaches have been used to characterize air quality at regional and urban scales [5–7]. However, there is a lack of accessible tools that can be easily applied to study near-source pollution and identify contributing sources and develop strategies for reducing emissions and exposure. Several studies have tackled the contribution of various sources to the local air quality using different approaches [8,9]. Monitoring-based assessments can characterize air quality in a community when enough monitors are deployed. However, understanding the relative contributions of emissions sources impacting the community would require modeling. Dispersion modeling can potentially provide information on relative contributions of emissions sources if adequate locally resolved emissions are available. However, local-scale emissions are typically the main source of uncertainty in modeling assessments. Also, model users require an intermediate to high level of technical expertise to develop community-scale emissions inventories, apply the models, and interpret results [10–12]. A combination of dispersion modeling and monitoring could be a practical approach for community-scale air quality characterization.

To address this need, the US EPA initiated research to develop an air quality modeling approach that combines dispersion modeling and measurements (including stationary, mobile measurements, and portable sensor technologies) to create accurate, fine-scale air quality characterization. The Kansas City TRansportation local-scale Air Quality Study (KC-TRAQS) was conducted over a one-year period to characterize air quality in residential communities located near major rail yards and assess the relative contributions of the rail yard and other emissions sources to particulate matter (PM_2.5_) and black carbon (BC) [13]. This large dataset with differing time and spatial resolutions requires innovative data analysis approaches to interpret and provide researchers and stakeholders with results that inform the extent and impact of air pollutants in the study area. In addition, innovative data analysis approaches are needed for emission source attribution for near-source exposure assessments. In this study, we combine dispersion modeling and measurements from the KC-TRAQS study. We use data fusion methods to combine observations from stationary and mobile measurements, and dispersion model estimates. Using these fused fields, we estimate relative source contributions of air pollution in the community.

## 2. Methods

### 2.1. KC-TRAQS Field Study

KC-TRAQS was a field measurement campaign designed to understand community air pollution on a local scale in an area affected by multiple sources, including major rail yards, freeways, distribution centers, and industrial facilities. The primary pollutants of interest were PM_2.5_ and carbon fraction of particulate matter. The main objective of this study was to characterize the spatial and temporal variability of air pollution in several neighborhoods in the southeast Kansas City area and to identify the impact of local air pollution sources on these selected neighborhoods. The study also tried to understand the influence of meteorological conditions and source activities such as trucking fleets and truck traffic, rail yards, passing railroad traffic, railyard maintenance activities on spatial patterns of air pollution. The KC-TRAQS study was conducted over a 1-year duration (from 24 October 2017 to 31 October 2018) and included site-specific meteorological data, measurement of multiple air pollutant species using various techniques such as usage of traditional sampling methods and instrumentation, lower-cost sensor packages, citizen science, and mobile measurement techniques, which are elaborated further below.

**Figure 1.**
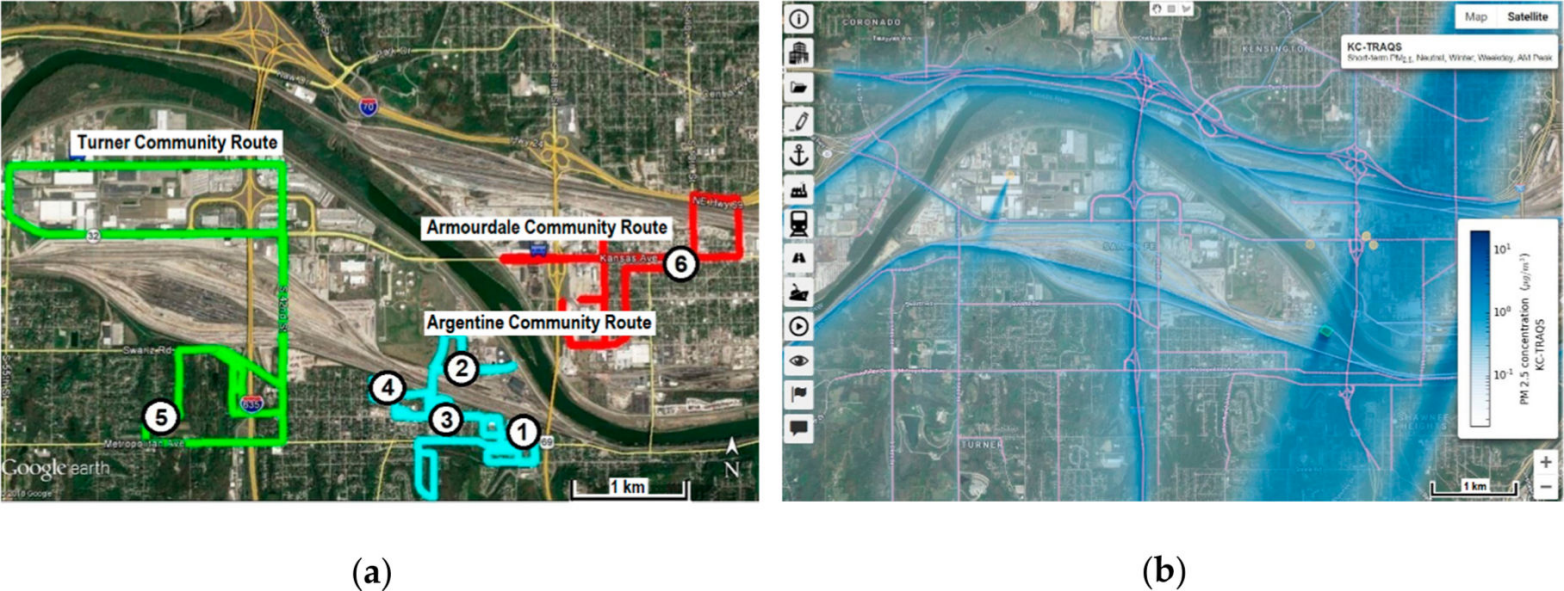
Locations of stationary and mobile measurements (**a**) and a screen shot of C-PORT model simulations of primary PM_2.5_ impacts (**b**) for selected meteorological conditions (winter, neutral stability, NNE winds).

The KC-TRAQS design included stationary site instruments to measure PM_2.5_ and BC in combination with laboratory analyses and mobile measurement techniques [13]. The stationary sites included traditional sampling methods (e.g., Federal Reference Method/Federal Equivalent Method (FRM/FEM)) and sensor packages (e.g., lower-cost sensors). The FRM/FEM sampling included PM_2.5_ gravimetric (i.e., Teflon) filters. Non-FRM/FEM sampling included quartz filters for measuring elemental and organic carbon (EC/OC) while the lower-cost sensor package included sensors streaming 1-min PM_2.5_ and BC data. Laboratory analyses were performed on the Teflon and quartz filters to obtain PM_2.5_ and EC/OC concentrations, respectively. In addition, Environmental-Beta Attenuation Monitors (E-BAMs) were included at selected fixed sites. The mobile monitoring utilized an instrumented electric vehicle recording 1-s ultrafine particulate matter number count (UFP), black carbon (BC), nitrogen dioxide (NO_2_), and carbon dioxide (CO_2_) data. Mobile monitoring used a combination of the Geospatial Monitoring of Air Pollution (GMAP) electric vehicle (Ford Focus) and a stationary sport utility vehicle (SUV; Ford Excursion) outfitted with fast-response air monitoring instruments which have been employed in previous studies [14]. The vehicles have separate, onboard solar and battery supplies powering the air monitoring instruments. Two mobile monitoring intensive studies were conducted from 10 October to 14 November 2017 and from 19 February to 18 March 2018. Driving routes for these intensive studies were chosen to complement the field sampling, with a total of ~40 sampling days, including 10–20 repetitions per day for each route. Sampling was limited to those days when the 24-h advanced weather forecast indicated that the chance of rain in the targeted route area was less than 30% during the target sample hours. The area of study was in SE Wyandotte County, KS and focused on the neighborhoods of Turner, Argentine, and Armourdale (Figure 1a). The study area includes part of the Kansas River, and the river valley that runs through SE Kansas City, KS and is part of the greater Kansas City, KS and Kansas City, Missouri urban area.

### 2.2. Dispersion Modeling to Support Field Study Design

Modeling was used to support field study design. The stationary measurement sites were selected based on local knowledge and past field campaign experience: 1—Police Station, 2—American Legion, 3—Fire Station, 4—Clopper Field, 5—Leo Alvey, and 6—Bill Clem, as shown in Figure 1a. In the design phase of the KC-TRAQS study, the “presumptive placement” of these fixed sites has been corroborated using the Community modeling system for near-PORT, or C-PORT [15]. C-PORT is a research-grade screening model designed to be an easy-to-use computer modeling and visualization tool for exploring the range of potential impacts of changes in emissions and meteorology on local-scale air quality. In this study, we used C-PORT to assess the spatial and temporal coverage of the measurement sites relative to the entire study area extent (Figure 1b). Mobile monitoring routes (Figure 1a) were also adjusted based on the results of model simulations. The C-PORT modeling system includes pre-loaded meteorological inputs (wind speed, *uStar*, *wStar*, convective mixing height, mechanical mixing height, *L*, surface roughness, and reference height) and allows the user to run the model online for selected meteorological conditions. The pre-loaded meteorological inputs are based on hourly weather measurements from the National Weather Service (NWS) monitoring site that is nearest to the study location for 2011. C-PORT allows the user to simulate air quality concentrations for any of the five representative meteorological conditions (Stable, Slightly Stable, Neutral, Slightly Convective, and Convective), for each season (Winter & Summer) [15]. We used C-PORT to simulate a range of pollutant concentrations (due to variations in meteorological conditions) expected to be seen at the monitor locations. This information is important for selecting types of air quality sensors, locations for sampling, and the frequency of the measurements. The initial KC-TRAQS field study design included various monitoring instruments with the sampling frequency ranging from seconds to daily averages. The modeling indicated potential impacts of stationary sources with narrow plume patterns in the study area, thus suggesting more frequent sampling.

The model was also used to simulate annual averages, based on 100 representative meteorological hours using hourly observations from the Kansas City (Missouri) Downtown Airport, located approximately 5 km east of the study area, within the same river valley as the three communities of interest. To compute annual averages, C-PORT uses the METeorologically weighted Averaging for Risk and Exposure (METARE) approach described in Chang et al. [16], wherein dispersion is modeled for 100 explicit hours and then a weighted average is computed using those explicit 100 hourly concentrations and the frequency of occurrence of those conditions during the entire year. The model predictions provided spatial patterns of pollutant concentrations likely to occur during the monitoring campaigns.

### 2.3. Dispersion Modeling to Support the Analysis

**Figure 2.**
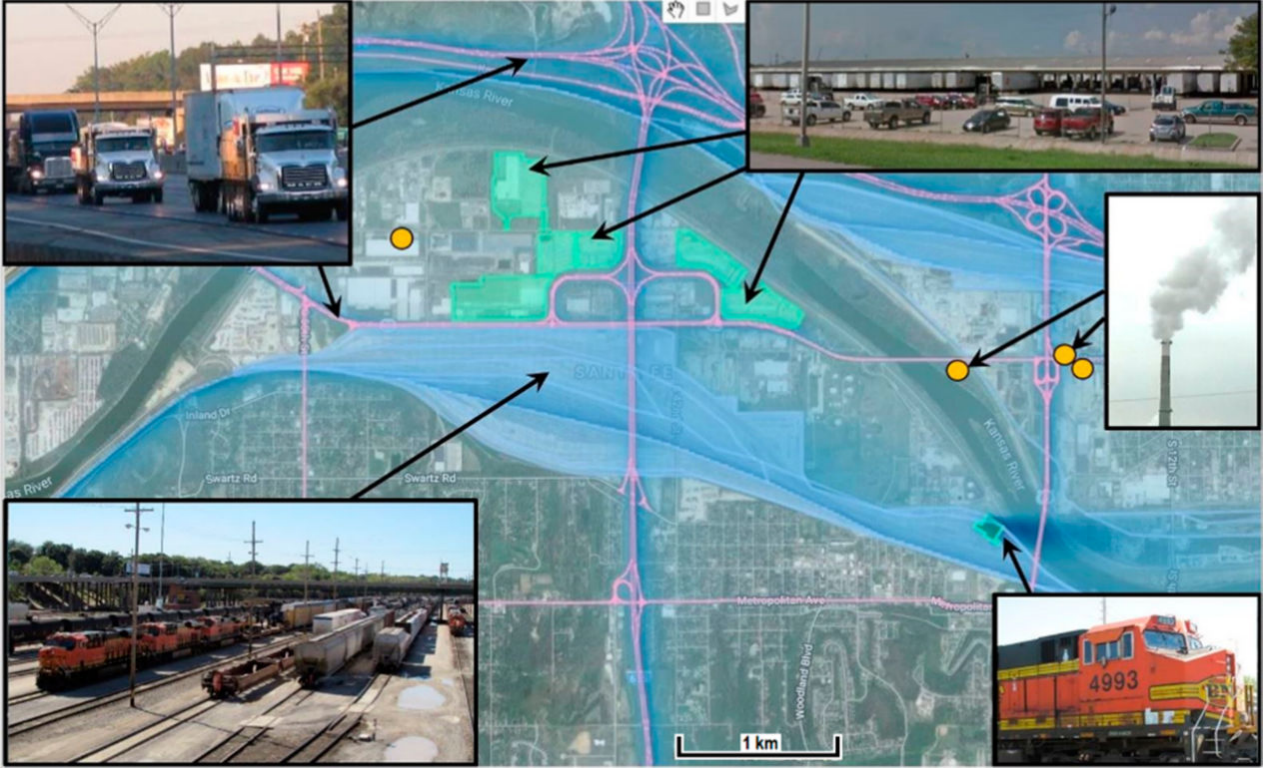
Map of the study area showing locations of emission sources and a screen shot of C-PORT model simulations of primary PM_2.5_ concentrations.

Modeling was also used to help interpreting field study data. We used a combination of dispersion modeling and monitoring data from KC-TRAQS to estimate the relative contribution of air pollution sources impacting the community such as roadway traffic, rail yards, warehouses, and stationary sources (Figure 2).

In the first phase of the analysis, we used C-PORT modeling tool to simulate air quality concentrations for selected groups of emission sources (roadways, rail, rail yards, warehouses, and industrial sources). Several scenarios were defined and simulated to estimate the relative contributions of the different source sectors to the air quality over the KC-TRAQS study domain. The model scenarios included simulations for roadway sources, rail sources, maintenance facility, and stationary sources, modeled as separate source groups. The simulations were conducted for typical meteorological conditions during winter and summer seasons, and for annual averages. For these simulations, the modeling domain encompassed the sources of interest and covered an area of approximately 10 km × 10 km with spatial resolution of 75 m (Figure 2). Sensitivity tests were performed to assess the impact of the meteorological conditions (atmospheric stability, wind direction) on the patterns of air pollutant concentrations, and to further identify the conditions that are responsible for worst-case pollution episodes. The sensitivity tests included simulations for neutral, slightly stable and stable, and slightly convective and convective conditions, for both winter and summer seasons. Simulations were conducted for various wind directions (northerly, southerly, and seasonally average) to analyze the potential impact of selected source categories under different meteorological conditions.

In the second phase, we conducted dispersion simulations for the entire period of the KC-TRAQS study—from October 2017 to October 2018. We used a dispersion model [15] based on algorithms for stationary and area sources from AERMOD [17] and algorithm for line sources is based on the analytical approximation for line sources [18], consistent with the US EPA research model for line sources R-LINE [19]. The model was optimized for computational efficiency for future inverse modeling applications. The model simulations used hourly meteorological observations from the Kansas City (Missouri) Downtown Airport. A local-scale emission inventory has also been developed for the KC-TRAQS study. Emission sources were categorized as on-road mobile sources, rail line sources, industrial point sources, and area sources (e.g., rail yards, large warehouses). Roadway emissions are based on a combination of road network, traffic activity, and emissions factors. The modeling approach for roadways emissions was consistent with the approach used in the C-LINE web-based model [20], where emissions for each road link are calculated by combining national database information on traffic volume and fleet mix with emissions factors from EPA’s MOVES modeling system. Model inputs for point source emissions from industrial activities and electric generating unit were based on EPA National Emissions Inventories (NEI)-2014 [21]. These point sources were processed using the Sparse Matrix Operator Kernel Emissions (SMOKE) modeling system v3.6.5 [22,23]. Using SMOKE’s toolset, point sources were reviewed and verified to have appropriate stack parameters. The default stack parameters were used when actual stack parameters were not available. The rail category includes emissions from railroad equipment, line haul locomotives and yard locomotives. Railroad emissions are allocated to railroads using ArcGIS and modeled as line sources, and rail yard emissions are allocated to rail yard polygons in ArcGIS modeled as area sources. Emissions from large warehouses and distribution centers in the modeling domain were also modeled as area sources. Rail yard polygons were drawn in the open-source geographic information system QGIS software. These are based on Google aerial imagery, and the polygons represent a best-guess at the boundaries of all rail yard-related activity. Emissions for the rail yard are based on 2014 NEI v2 values for Source Classification Code (SCC) 28500201 as shown in Table 1. For the Argentine Yard, a separate polygon was drawn to represent the maintenance facility (MF) shed. Since the NEI emissions estimates for the Argentine Yard included the MF shed, it was necessary to find a way to distribute the NEI emissions value between the maintenance shed and the emissions from the rest of the rail yard. Based on the 2015 Argentine Yard Emission Inventory [24], maintenance activity is a substantial fraction of the total emissions from the yard. E2 Managetech provided estimates for NO_x_ and PM_2.5_, but the study did not include EC_2.5_. However, the E2 Managetech study found a consistent EC/PM_2.5_ ratio for all the railyards, where EC_2.5_ is 0.7712 times the PM_2.5_ value. For this study, we assumed that all maintenance activity at the Argentine Yard occurred in the MF shed.

Warehouse facility polygons were also drawn in QGIS based on Google aerial imagery, and the polygons represent the footprint of the entire property. To determine the emissions from each facility, the QGIS measure tool was used to measure the area in square feet of the footprint of the building(s) inside the warehouse facility polygon. Based on methodology used by Michael Baker Jr. Inc. in a 2007 report for PA DEQ Bureau of Air Quality [25], the number of trucks serving the facility on a daily basis was estimated as 0.21 times the square footage/1000. The total trucks were then allocated into six different minutes idling bins (0–4; 5–9; 10–14; 15–19; 20–24; 25–29), using the same assumptions that were used in the Michael Baker Jr, Inc. report [25]. These daily idling bins were then multiplied by 250 work days in a year to get annual idling minutes by bin.

The emission rate per hour values for hoteling (idling) trucks from MOVES 2014v2 Platform for SCC code 2202620153 (Extended Idle) were used as base emission rates. For January, the value corresponding to 30 degrees F was used, because the mean temperature in January is 29.3 degrees according to Annual Climatology for Kansas City, MO from the National Drought Mitigation Center [26]. Similarly, for July, the value corresponding to 80 degrees F was used, because the mean temperature in July is 81.3 degrees F. The total number of hours idling for each facility was then multiplied by the rates per hour listed above and then multiplied by 0.00965625 to convert to tons per year. The January and July emissions were then averaged to estimate the annual emissions in tons/year.

Emissions for nine large area sources were simulated: 1—Armstrong Rail Yard, 2—Associated Wholesale Grocers, 3—USPS Distribution Center, 4—BNSF Maintenance Facility, 5—UPS Freight, 6—Sam’s Club Distribution, 7—Estes Express Lines, 8—Union Pacific Armourdale Rail Yard, and 9—Santa Fe Argentine Rail Yard. A summary of emissions for NO_x_, PM_2.5_ and its EC_2.5_ components is shown in Table 2.

### 2.4. Data Fusion

Using dispersion modeling to estimate relative contributions of emissions sources requires adequate locally resolved emissions as model inputs. Often, community-scale emissions inventories are not available for model applications and developing such locally resolved emissions is typically resource intensive. Recent development in monitoring technology has improved the performance of air quality sensors [27]. These monitors can help to detect local hot spots and thus provide significant information for local-scale air quality characterization [28,29]. However, because these sensors are usually irregularly spaced and often used as a part of special studies, there is a need to combine the sensor data with modelling techniques in order to construct air pollution concentration maps at the community scale. A data fusion method based on the Bayesian Maximum Entropy (BME) method of modern spatiotemporal geostatistics has been applied for characterizing PM_2.5_ at regional scales [30] and for ozone [31]. In this study, we applied the BME-based data fusion method for the KC-TRAQS modeling domain to create a spatial map of BC concentrations. The BME framework is as follows. Let boldface represent vectors of values, upper case represents random values and lower case represents deterministic (e.g., observed) values. Using this notation, let *s* be a spatial location, *X(s)* be a Spatial Random Field (SRF) depicting a random value at location *s*, *z*_*h*_ be the vector of 27 observed values of annual EC concentration at the 6 stationary sites and 21 mobile monitoring zones, and *o*_*h*_ be offset values consisting of the dispersion model values at these 27 points. First, we calculate the residual concentrations *x*_*h*_ = *z*_*h*_ − *o*_*h*_. We define *X*(*s*) as a homogeneous (i.e., constant mean) SRF representing the variability of residual concentrations *x*_*h*_. We model the covariance of *X*(*s*) by fitting an exponential covariance model through the experimental covariance values obtained from the *x*_*h*_ data. We then obtain the BME estimate *x*_*k*_ of the residual concentration at some unsampled location *s*_*k*_ by interpolating the *x*_*h*_ observed data using the BMElib numerical library [32,33] with a general knowledge consisting of a constant mean and the covariance of *X*(*s*), and site-specific knowledge consisting of the observed values *x*_*h*_ treated as hard (i.e., exact) data. Finally, the dispersion model value *o*_*k*_ at location *s*_*k*_ is added to *x*_*k*_ to obtain the estimate *z*_*k*_ of annual EC concentration at the estimation location **s**_k_. In this case, BME reduces to BME Ordinary kriging, and the estimate *z*_*k*_ is a fusion of the model and observed values. The BME-based data fusion method has been applied to create concentration maps in similar studies [34,35].

## 3. Results

### 3.1. Combining Stationary and Mobile Monitoring Data

**Figure 3.**
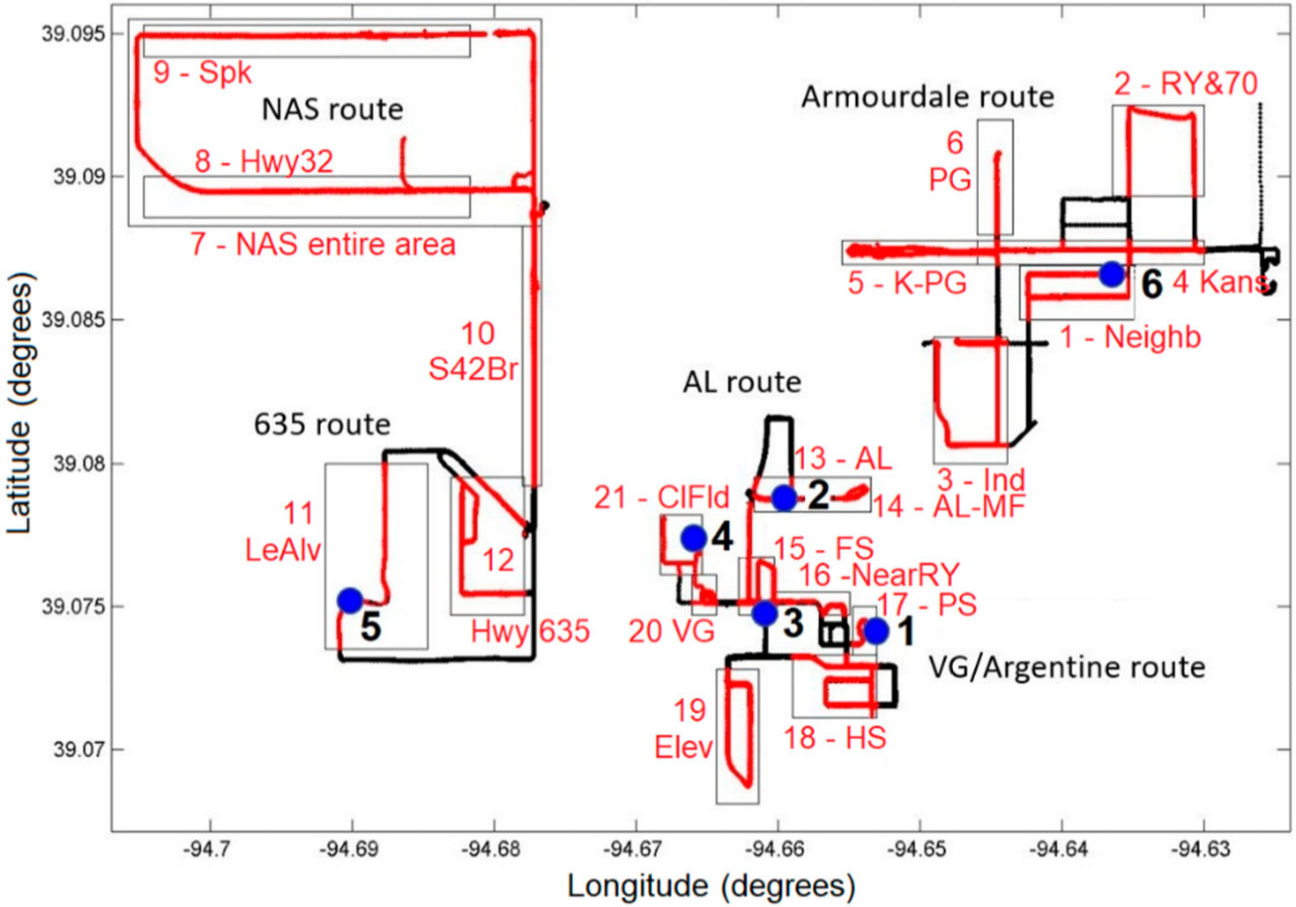
Schematic map of the study area showing mobile monitoring routes and selected sampling areas.

Air quality observations at six monitoring sites using lower-cost sensor packages provided a year-long time series of air pollutant concentrations, thus providing temporal resolution of air quality in the study area. Mobile monitoring provided additional spatial coverage during two intensive studies with a total of ~40 sampling days, including 10–20 repetitions per day for each route. The mobile monitoring study design included five broad sampling areas: Northern Area sources (NAS) area, Highway 635 area, American Legion (AL) area, Village Green (VG) & Argentine area, and Armourdale area. Based on the analysis of the mobile monitoring data in these five sampling areas, we selected more narrower “air quality zones” within the five sampling areas, representing areas expected to be impacted by different sources based on evaluation of the study area and modeling results. We identified 21 air quality zones: 1—Armourdale neighborhood (Neigb), 2—Armourdale rail yard & Hwy 70 (RY&70), 3—Armourdale industrial area (Ind), 4—Kansas Avenue (Kansas), 5—Kansas Avenue & PG facility (K-PG), 6—near PG facility (PG), 7—Northern Area Sources Route entire area (NAS), 8—Highway 32 (Hwy32), 9—Speaker Rd (Spkr), 10—State route 42 Bridge (S42Br), 11—Leo Alvey area (LeAlv) 12—Highway 635 (635), 13—American Legion area (AL), 14—near Maintenance Facility (AL-MF), 15—Fire Station (FS), 16—near Rail Yard (NearRY), 17—Police Station (PS), 18—Argentine High School (HS), 19—Argentine elevated area (Elev), 20—Village Green (VG), 21—Clopper Field (ClFld), as shown in Figure 3. These air quality zones were selected to be representative of the areas impacted by different sources. The zone selection was made based on examining the data after it was collected and examining spatial imagery for the region as shown in Google maps, the prevalent meteorological patterns, and nearby sources. For example, the air quality zone next to the stationary site 2 (AL site), is intended to capture the impact of the Maintenance Facility (MF) of a nearby rail yard.

The routes are designed to provide spatially resolved pollutant concentration measurements in and around the three communities, as well as six fixed measurement sites in the area as described by Kimbrough et al. [13]. During the Fall 2017 and Spring 2018 monitoring campaigns, the GMAP vehicle was driven continuously along one of five pre-determined routes as shown in Figure 3 (Turner community route, I-635 route, American Legion route, Argentine community route, Armourdale community route). Vehicle battery allowed for approximately 6 to 10 h of continuous sampling each day, allowing for multiple laps per session. Sampling was done for multiple days, with a larger frequency of measurements in the Argentine community to better evaluate emission impacts from the rail yard and maintenance facility.

**Figure 4.**
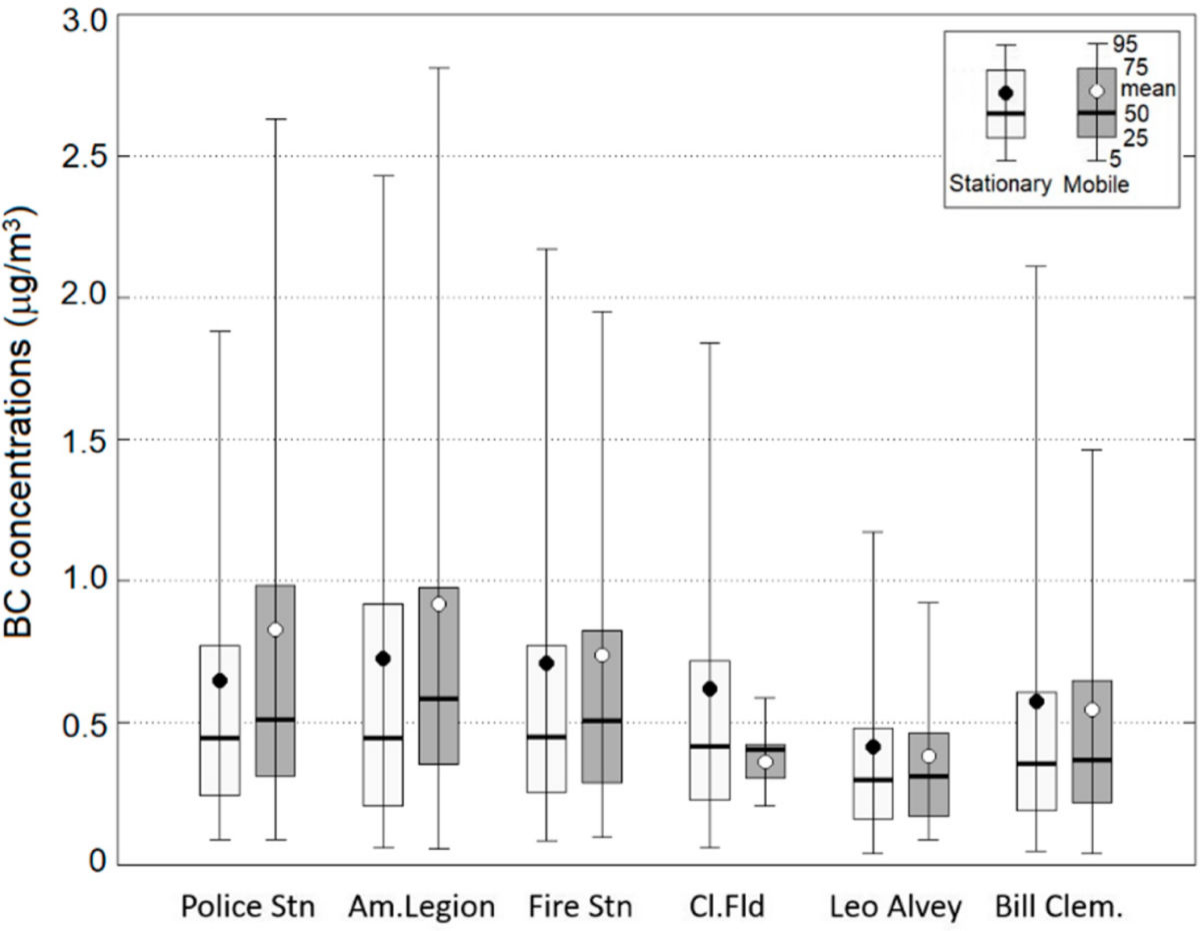
Comparison of distributions of observed BC concentrations at six stationary sites and at selected mobile monitoring areas representing concentrations around the six stationary sites.

A comparison of distributions of observed BC concentrations at six stationary sites and at selected mobile monitoring areas representing concentrations around the six stationary sites is shown in Figure 4. As can be seen from the figure, median values of observed concentrations from mobile monitoring compare well with median concentrations at the stationary sites. The 25–75 percentile intervals from mobile monitoring also compare well with those at the stationary sites. Thus, it gives us more confidence that mobile monitoring data have better spatial coverage and can potentially identify hot spot areas not captured by the stationary monitoring network.

**Figure 5.**
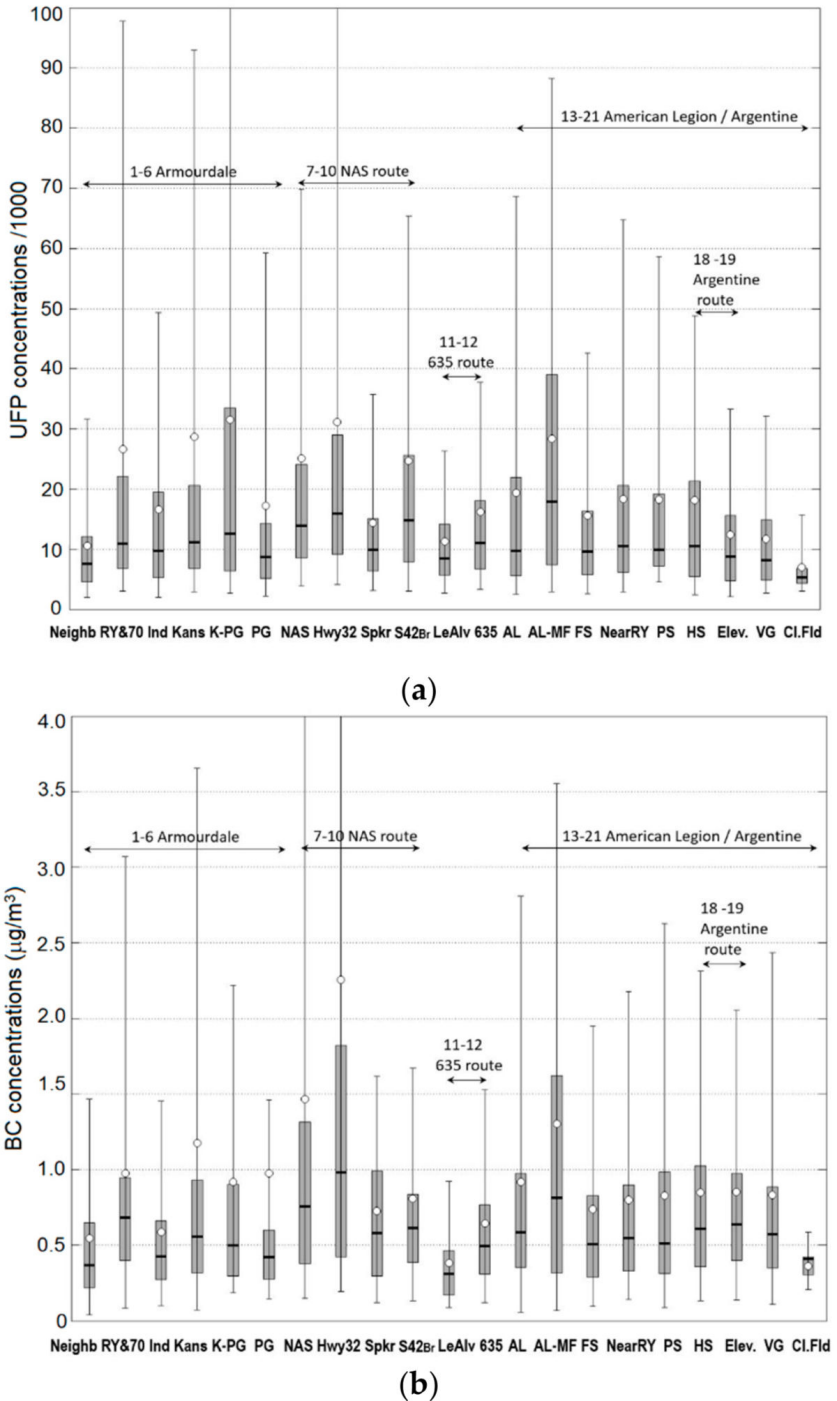

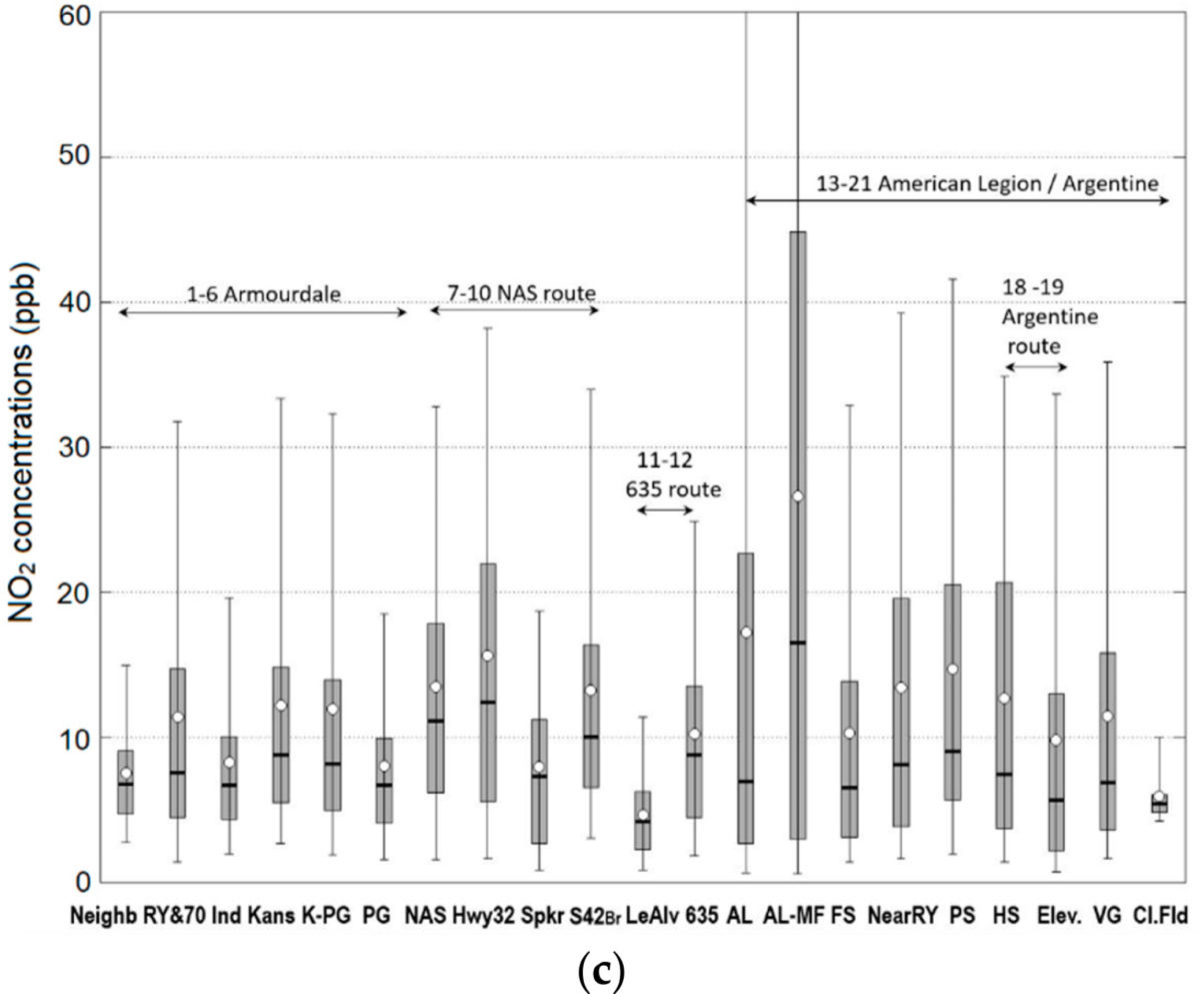
Distributions of observed concentrations of UFP (**a**), NO_2_ (**b**), and BC (**c**) at the selected 21 mobile monitoring air quality zones.

Using the “air quality zone” approach for processing the mobile monitoring data, we extended the limited set of six stationary sites with additional 21 pseudo-stationary sites, as shown in Figure 3. Distributions of observed concentrations of UFP, NO_2_ and BC at the selected 21 air quality zones from mobile monitoring are shown in Figure 5. From the comparison of distributions at various zones in the American Legion area, it is apparent that AL-MF has higher levels of concentration for both NO_2_ and BC, thus indicating the potential impact of the MF. The Hwy32 zone also shows higher mean values of the distributions for all pollutants, indicating the potential impact of warehouses in the NAS area. However, roadway traffic emissions could also contribute to the distributions of concentrations at the Hwy32 mobile monitoring zone.

### 3.2. Combining Dispersion Modeling to Estimate Relative Contribution of Air Pollution Sources

**Figure 6.**
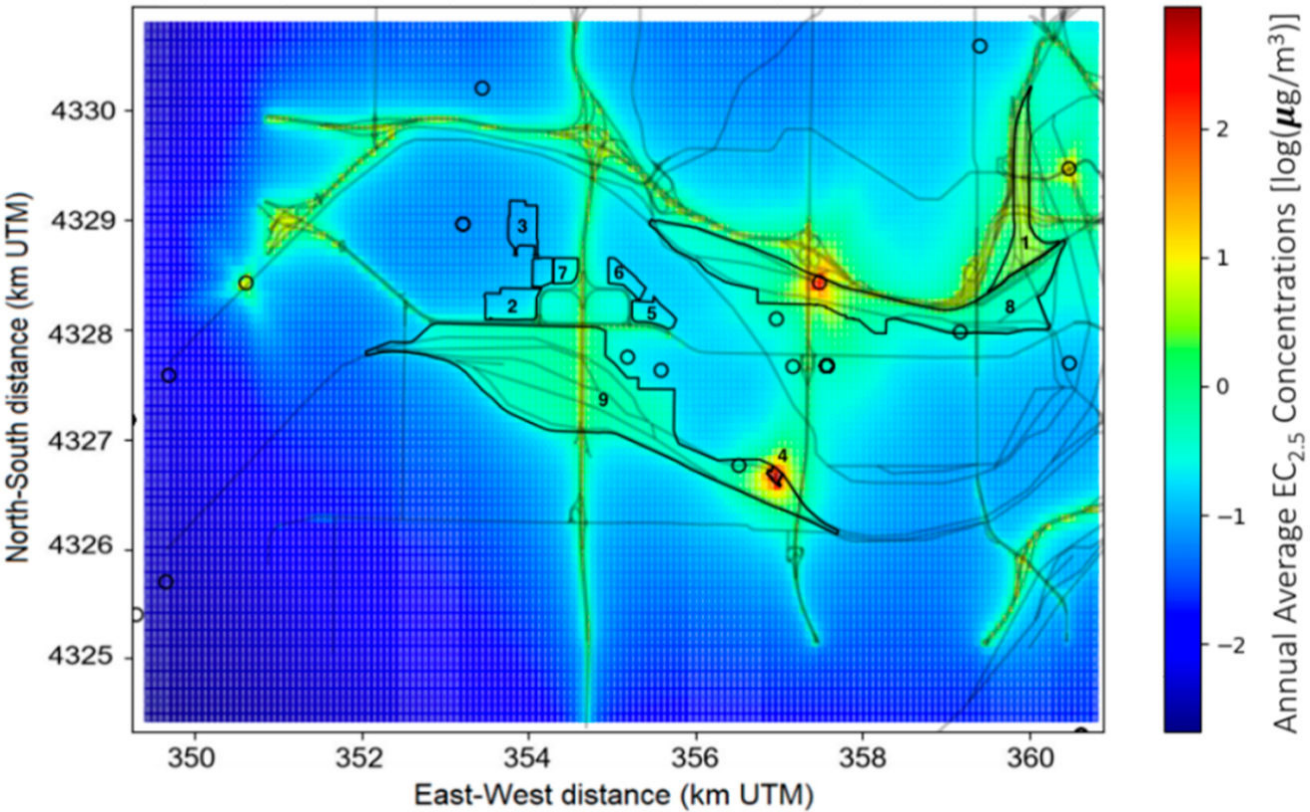
Modeled annual average EC_2.5_ concentrations. Note: numbers indicate locations of 9 main area sources (1—Armstrong Rail Yard, 2—Associated Wholesale Grocers, 3—USPS Distribution Center, 4—BNSF Maintenance Facility, 5—UPS Freight, 6—Sam’s Club Distribution, 7—Estes Express Lines, 8—Union Pacific Armourdale Rail Yard, and 9—Santa Fe Argentine Rail Yard). Concentrations are shown in logarithmic scale.

Dispersion model simulations were conducted to estimate relative contributions of various source sectors to outdoor air quality concentrations, including roadway traffic, rail yards, warehouses, and industrial sources that potentially affect the neighborhoods of Turner, Argentine, and Armourdale. A map of modeled ground-level annual average EC concentrations is shown in Figure 6. The model receptors are distributed in a 75-m-spaced uniform grid to capture sharp gradients of pollutant concentrations near roadways and other emission sources in the modeling domain. The map shows higher concentrations near major roadways and near the rail yard. It also shows elevated levels of pollutant concentrations near some stationary sources within the rail yards and near MF.

**Figure 7.**
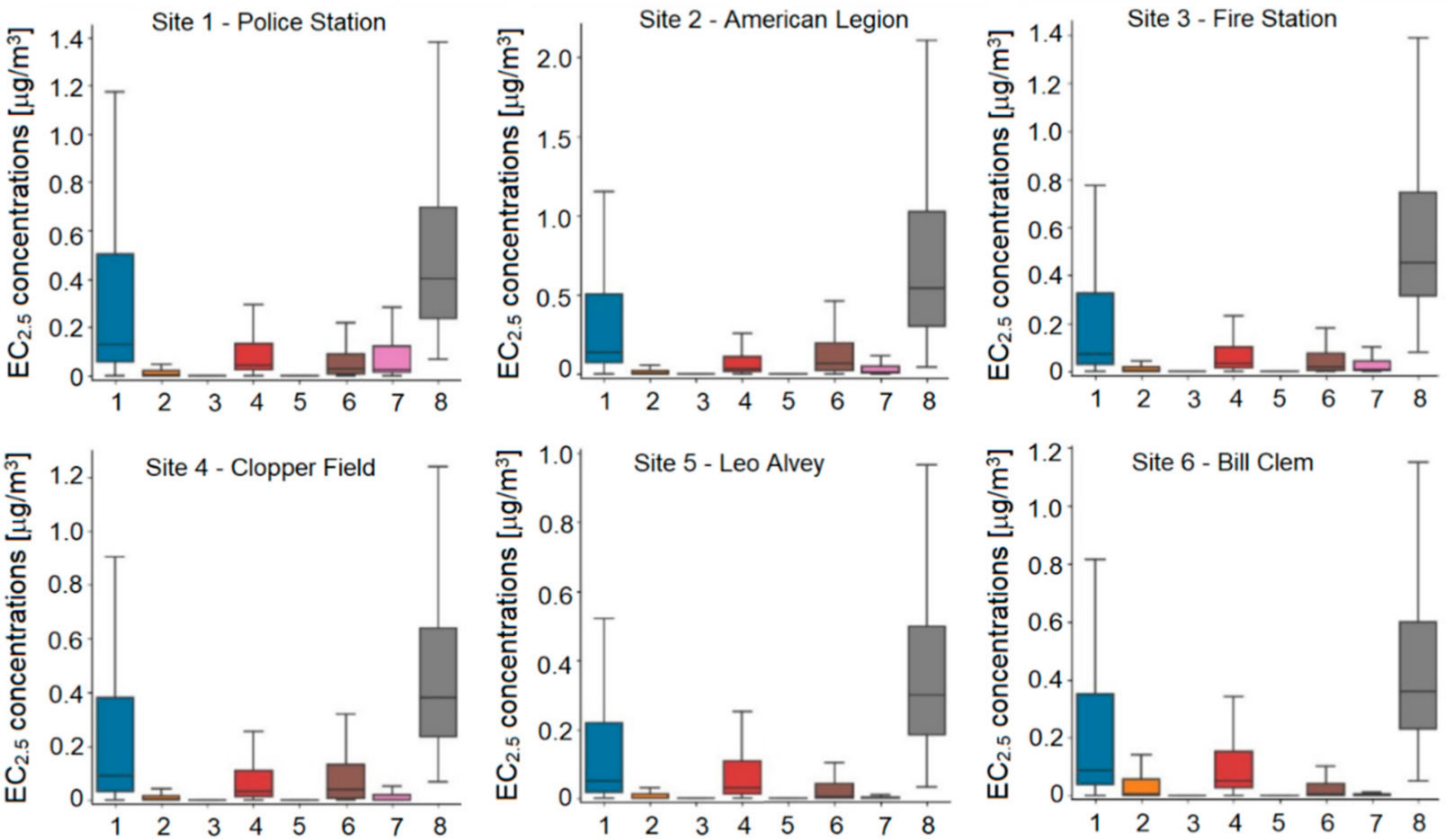
Comparison of distributions of modeled EC_2.5_ concentrations at six monitoring locations for different groups of sources (1—All sources, 2—Point sources, 3—Rail, 4—Roads, 5—Maintenance Facility, 6—Rail Yards, 7—Warehouses) and observations (indicated as group 8). Note: The line in the box indicates the median, box extents indicate the interquartile range (IQR), and whiskers indicate 1.5 times the IQR. Data beyond the whiskers were excluded.

The modeling also provided information to help understand the relative contribution of various sources impacting the communities in the KC-TRAQS study area. The main source of primary particulate matter emissions is assumed to be the rail yard, which includes emissions from line-haul locomotives, switching locomotives operating in the rail yard, and locomotive maintenance facility. However, roadway traffic is also a significant source of particulate matter emissions. Particulate matter can also be transported into the study area from different industrial sources. Warehouses and distribution centers can also contribute to the total PM concentrations in the study area. Thus, understanding relative contributions of various emissions sources using measurements alone is quite challenging. Dispersion modeling by design provides concentration estimates for each source modeled and for all model receptors. To illustrate the source contribution analysis based on dispersion modeling we focus on EC_2.5_ component of primary particulate matter. Figure 7 shows distributions of modeled EC_2.5_ concentrations at six monitoring sites: 1—Police Station, 2—American Legion, 3—Fire Station, 4—Clopper Field, 5—Leo Alvey, and 6—Bill Clem. The distributions are based on hourly modeled predictions for the entire study period from October 2017 to October 2018. The figure also shows distributions of modeled concentrations for several selected source categories: 1—All sources, 2—Point sources, 3—Rail, 4—Roads, 5—Maintenance Facility, 6—Rail Yards, 7—Warehouses. Distributions of observed EC_2.5_ concentrations are also included for comparison purposes. While the model generally predicts lower concentrations than observed at all six monitoring sites, the model predictions for specific source groups provide some insight on contributing sources. For example, the rail yard contribution is higher at sites 2 (American Legion) and 4 (Clopper Field), which are closest to the rail yard, and lower at other sites. The model predicts a relatively higher impact of the locomotive maintenance facility at site 2 (American Legion), but the overall impact is low. Model predictions indicate a higher impact of industrial point sources at site 6 (Bill Clem) than at other sites. Roadway emissions equally contribute to model concentrations at other sites with a slightly higher contribution at site 6 (Bill Clem).

### 3.3. Application of a Data Fusion Method Using Dispersion Modeling and Observations

**Figure 8.**
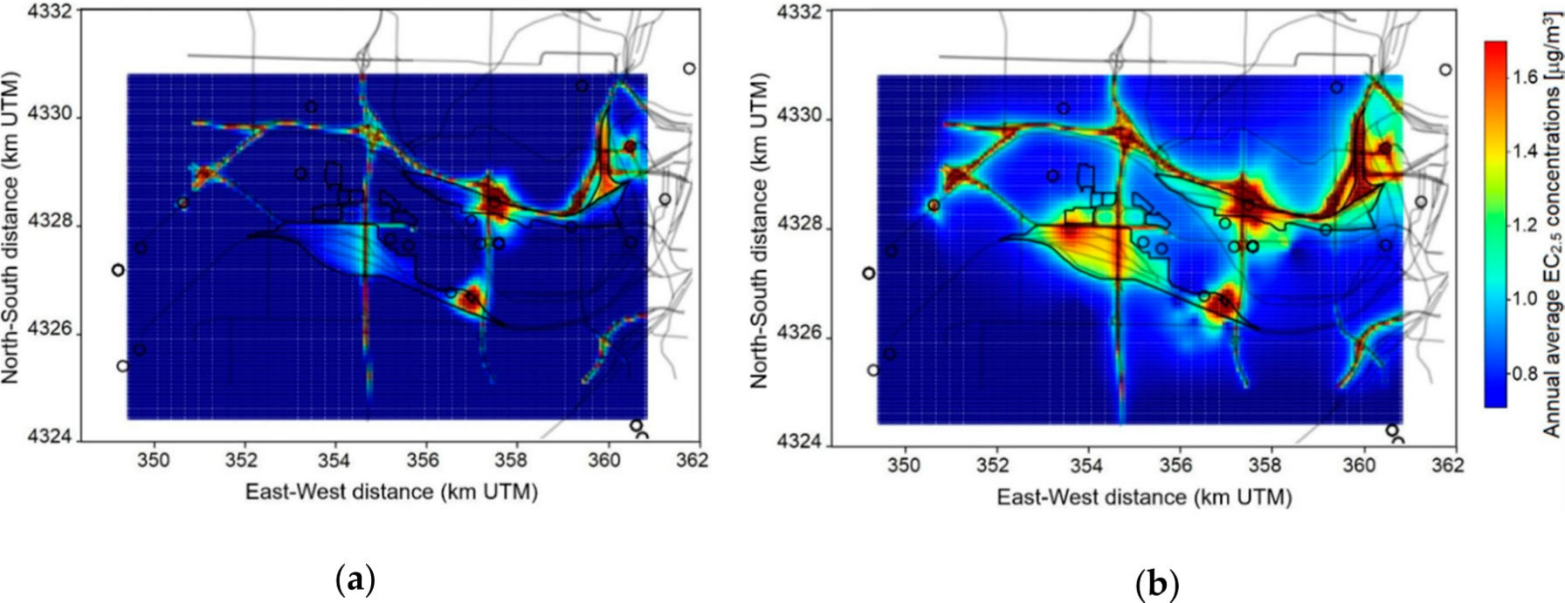
Map of EC_2.5_ concentrations based on dispersion modeling (**a**) and based on data fusion (**b**).

We applied the BME-based data fusion method for the KC-TRAQS study area using observations from stationary and mobile monitoring and dispersion model results. In this application, we used a publicly available software of Modern Spatiotemporal Geostatistics implementing the BME theory [36]. The results of the data fusion application are shown in Figure 8. The figure compares spatial maps of annual average EC_2.5_ concentrations based on dispersion model simulations (Figure 8a) and based on the data fusion. The figure shows similar patterns of pollutant concentrations in both panels and indicates areas of elevated concentrations near main emission sources (roadways, rail, industrial point sources). However, the data fusion method provides higher concentrations near these sources because the data fusion method corrects the concentrations using observations at six stationary sites and 21 mobile monitoring zones. The data fusion method also shows different spatial patterns. Specifically, it shows an area with elevated pollutant concentrations near warehouses in the NAS area and shows a larger hot spot near MF of a nearby rail yard. Thus, the data fusion method provided information that could help improve emissions inputs for dispersion modeling.

The model performance was evaluated using quantitative model performance measures [37]: mean bias (MB), mean error (ME), root-mean-squared error (RMSE), and normalized mean bias (NMB), defined as:

(1) $$MB=\frac{1}{n}\sum_{i=1}^{n}\left(C_{i}^{M}-C_{i}^{O}\right)$$

(2) $$ME=\frac{1}{n}\sum_{i=1}^{n}\left|C_{i}^{M}-C_{i}^{O}\right|$$

(3) $$RMSE=\sqrt{\sum_{i=1}^{n}{\left(C_{i}^{M}-C_{i}^{O}\right)}^{2}/n}$$

(4) $$NMB=\sum_{i=1}^{n}\left(C_{i}^{M}-C_{i}^{O}\right)/\sum_{i=1}^{n}C_{i}^{O}$$

where $C_{i}^{M}$ is modeled annual average concentration at the site *i*, $C_{i}^{O}$ is observed annual average concentration at the site *i*, and *n* = 27 is a total number of sites in the study area (6 stationary monitors plus 21 “air quality zones” from mobile monitoring). A comparison of performance measures for dispersion modeling and data fusion-based concentration estimates is given in Table 3. As can be seen from the table, the data fusion method helped to improve model predictions by reducing both bias and error metrics.

## 4. Summary and Conclusions

This study demonstrates the use of dispersion modeling to support the design of air monitoring field studies. Typically, monitoring field studies and modeling assessments are conducted separately. Model simulations are usually performed independently, using available emissions inventories as model inputs and then, model predictions are evaluated against observations from routine monitoring networks or from special field studies. In this study, we used dispersion modeling in support of the KC-TRAQS field study design to evaluate and adjust placement of fixed monitoring sites and mobile monitoring routes.

Determining the location of the most appropriate measurement sites for a field measurement campaign utilizes a combination of subjective factors including field experience, knowledge of an area, engineering judgement and project goals. While these subjective factors may be supplemented by meteorological data, topographic data and locating known anthropogenic emission sources, a more rigorous approach is advantageous when an area has multiple complex factors. A more rigorous approach (i.e., modeling) can help explain subjective factors and provide confirmation that the siting criteria were applied correctly. Modeling can also provide an indication of the appropriate spatial coverage of measurement sites. The KC-TRAQS study area had factors that contributed to the complexity of locating appropriate measurement sites to achieve project goals. These factors included the complex meteorological conditions such as local inversions and wind flow changes due to complex topography of a river valley surrounded by rolling hills. In addition, the area has a combination of emission sources including residential, light industrial and commercial facilities, and transportation sources. These factors, including meteorology and emissions, were used to confirm the presumptive placement of stationary measurement sites for KC-TRAQS [13]. The measurements frequency is a critical element of the field study design. The initial KC-TRAQS field study design included various monitoring instruments, with the sampling frequency ranging from seconds to daily averages. The modeling indicated potential impacts of stationary sources with narrow plume patterns in the study area, thus suggesting more frequent sampling (e.g., 1-s or 1-min as compared to 24-h average sampling time). Mobile monitoring was used in the KC-TRAQS study to identify spatial patterns of pollutant concentrations in the study area. We used modeling to evaluate the initially designed mobile monitoring routes and adjusted them based on the modeling results. For example, the Village Green route was expanded to the west to cover the Clopper Field area due to potential impacts from the modeling results. This modeling also indicated higher concentrations near Hwy 32 due to truck traffic, therefore the mobile monitoring routes were adjusted. Also, the I-635 route and NAS route were treated as separate sampling areas. Modeling also helped to optimize sampling schedule—more sampling days in areas where modeling indicated elevated levels of pollutant concentrations.

Dispersion modeling can also provide estimates of relative contributions of emissions sources potentially impacting the community. Thus, dispersion modeling can be used for planning future mitigation. Because dispersion models explicitly relate source strength to air quality, models can be used to predict changes in the concentration field when emissions change. However, dispersion modeling applications require adequate locally resolved emissions as model inputs. At community scales, such inventories are often unavailable. Developing local-scale inventories is usually resource intensive, but can add value to the study. Finally, local-scale emissions are typically the main source of uncertainty in modeling assessments. A combination of dispersion modelling and monitoring could be a practical approach for reducing uncertainty in community-scale air quality modeling applications.

In this study, we show an illustrative example of an air quality modeling approach that combines dispersion modeling and measurements (including stationary and mobile measurements) to create more robust, fine-scale air quality characterization in support of the KC-TRAQS study, designed to evaluate air pollution in a community affected by major transportation sources in Kansas City, Kansas. We first applied a dispersion model using a readily available emissions inventory. Then, we combined the model predictions with monitoring data (including observations from a limited number of monitoring sites and mobile monitoring) using a data fusion approach. A variety of data fusion methods are available. In this study, we used the BME method [30] to combine observations from stationary monitors, observations from mobile monitoring, and results from dispersion modeling to characterize air quality in the study area. The results demonstrate that mobile monitoring could help to provide better spatial coverage for the study area. In the KC-TRAQS application, we extended a limited set of six stationary sites with additional 21 pseudo-stationary sites from the mobile monitoring. The data fusion method was used to combine these two datasets with different time resolutions to create spatial maps of annual average pollutant concentrations for the entire study area. We also applied the data fusion method to combine the fused field of observations and dispersion model estimates. This data fusion application provided a spatially resolved map of air quality, which helped us to identify hot spots in the study area and improve emissions inputs for dispersion modeling applications to identify contributions from local and regional air pollution sources.

The next step is to adjust the model inputs, which can be done by either using inverse modeling techniques or by using an iterative approach, manually adjusting emissions. The improved model inputs would likely reduce uncertainty in model predictions and thus help the analysis of monitored data in the communities of interest. Future work will focus on developing inverse modeling techniques.

## Figures and Tables

**Table 1.** Emissions (tons/year) for the Argentine rail yard and maintenance facility.

Pollutant	Emissions from NEI-2014-v2 ^1^	MF Fraction %	MF Contribution	Railyard Contribution
NO_x_	158.218	44%	69.616	88.602
PM_2.5_	3.921	49%	1.921	2.000
EC_2.5_	3.024	49%	1.482	1.542

1 Emissions for Source Classification Code (SCC) 28500201.

**Table 2.** Emissions [tons/year] for nine large area sources were simulated.

n ^1^	Area Source Name	NO_x_	PM_2.5_	EC_2.5_
1	Armstrong	39.899	1.089	0.840
2	Associated Wholesale Grocers	1.061	0.0052	0.0012
3	USPS Distribution Center	0.384	0.0052	0.032
4	BNSF Maintenance Facility	69.616	1.921	1.482
5	UPS Freight	0.164	0.0022	0.0005
6	Sam’s Club Distribution	0.125	0.0017	0.0004
7	Estes Express Lines	0.068	0.0009	0.0002
8	Union Pacific Armourdale Yard	22.277	0.606	0.467
9	Santa Fe Argentine Yard	88.602	2.000	1.542

1 Numbers indicate locations of area source as shown in Figure 3.

**Table 3.** Comparison of performance measures for dispersion modeling and data fusion.

Statistical Measure	Dispersion Modeling	Data Fusion
MB	−0.415	−0.006
ME	0.433	0.282
RMSE	0.517	0.358
NMB	−52.9	−1.41
